# Response of the gut microbiota during the *Clostridioides difficile* infection in tree shrews mimics those in humans

**DOI:** 10.1186/s12866-020-01943-z

**Published:** 2020-08-20

**Authors:** Wenpeng Gu, Wenge Li, Wenguang Wang, Dexuan Kuang, Wenzhu Zhang, Caixia Lu, Na Li, Pinfen Tong, Yuanyuan Han, Xiaomei Sun, Jinxing Lu, Yuan Wu, Jiejie Dai

**Affiliations:** 1 Center of Tree Shrew Germplasm Resources, Institute of Medical Biology, Chinese Academy of Medical Sciences and Peking Union Medical College, Yunnan Key Laboratory of Vaccine Research and Development on Severe Infectious Diseases, Yunnan Innovation Team of Standardization and Application Research in Tree Shrew, Zhao zong Road 66, Kunming, 650118 China; 2 Department of Acute Infectious Diseases Control and Prevention, Yunnan Provincial Centre for Disease Control and Prevention, Kunming, 650022 China; 3 State Key Laboratory of Infectious Disease Prevention and Control, Collaborative Innovation Center for Diagnosis and Treatment of Infectious Diseases, National Institute for Communicable Disease Control and Prevention, Chinese Center for Disease Control and Prevention, Chang bai Road 155, Chang ping District, Beijing, 102206 China

**Keywords:** *Clostridioides difficile*, Tree shrew, Gut microbiota

## Abstract

**Background:**

*Clostridioides difficile* is a major cause of antibiotic associated diarrhea. Several animal models are used to study *C. difficile* infection (CDI). The tree shrew has recently been developed as a model of primate processes. *C. difficile* infection has not been examined in tree shrews. We infected tree shrews with hyper-virulent *C. difficile* strains and examined the alterations in gut microbiota using 16S rRNA gene sequencing.

**Results:**

*C. difficile* colonized the gastrointestinal tract of tree shrew and caused diarrhea and weight loss. Histopathologic examination indicated structures and mucosal cell destruction in ileal and colonic tissues. The gut microbial community was highly diversity before infection and was dominated by Firmicutes, Fusobacteria, Bacteroidetes, and Proteobacteria. Antibiotic administration decreased the diversity of the gut microbiota and led to an outgrowth of *Lactobacillus.* The relative abundance of Proteobacteria, *Gammaproteobacteria*, *Enterobacteriales*, *Lachnospiraceae*, *Enterobacteriaceae*, *Escherichia*, *Blautia,* and *Tyzzerella* increased following *C. difficile* infection*.* These taxa could be biomarkers for *C. difficile* colonization.

**Conclusions:**

In general, the disease symptoms, histopathology, and gut microbiota changes following *C. difficile* infection in tree shrews were similar to those observed in humans.

## Background

*Clostridioides difficile* is a major cause of antibiotic associated diarrhea in patients after hospitalization and antibiotic administration [1]. *C. difficile* infection (CDI) caused by toxigenic isolates can result in a wide range of outcomes, including asymptomatic colonization, diarrhea, life threatening pseudomembranous colitis, or intestinal obstruction [2]. CDI is the most common cause of healthcare-associated diarrhea and colitis, and is responsible for significant morbidity and substantial healthcare costs worldwide [3, 4]. The primary pathogenic mechanism of *C. difficile* is the production of enterotoxin A (TcdA) and cytotoxin B (TcdB); some *C. difficile* strains can produce a binary toxin, encoded by the *cdtA* and *cdtB* genes, that increases pathogenesis [5]. Since emerging in North American in 2005, hyper-virulent *C. difficile* isolates (PCR ribotype RT027) have caused several infections and outbreaks in the United States of America, Canada, and most European countries [6]. Recently, *C. difficile* RT078 has been reported from both hospitalized patients and environmental surfaces in eastern China [7]. This emergent strain also causes severe disease, especially in elderly patients [8].

Several animal models have been used to study different aspects of CDI, including pathophysiology, colonization, transmission, recurrence, and the impact of strain variability [9]. Small animals, such as mice, hamsters, and rats, as well as larger animals, such as foals, gnotobiotic piglets, and rhesus monkeys have been used to study CDI [10]. Each laboratory animal has its own application limitations and does not fully reflect the entire pathophysiological process of *C. difficile* infection in humans. For example, primates were rarely used due to their ethical restrictions; the lesion sites of rodents infected by *C. difficile* were different from human patients; germfree animals didn’t really show the ecological niche of human intestine. The utility of the animal model mainly depends on the similarities in pathological characteristics with humans. The tree shrew (*Tupaia belangeri*) belongs to the family Tupaiidae and order Scandentia, and is widely distributed in South and Southeast Asia [11]. Compared with rodents, tree shrews were considered as an alternative animal model for non-human primates. Simultaneously, use of the tree shrew as a laboratory animal has increased in recent years due to its low cost of maintenance, life span, small body size, short reproductive cycle, and close relationship to primates [12]. Several human diseases models have been constructed by using this animal [13]. In view of advantages of tree shrews mentioned above, we attempted to use this animal as a novel model for CDI, compared the characteristics of infection with other animals, and explored its feasibility and applicability.

In addition, disruption of the gut microbiota is another important component of CDI [14]. A number of animal models have been developed to examine the relationship between the gut microbial community and *C. difficile* infection [15, 16]. However existing models do not fully capture the relationship between the human gut microbiota and *C. difficile* pathogenesis. Therefore, we further analyzed the gut microbiota changes of tree shrews during CDI process by using 16SrRNA gene sequencing in this study. The features of intestinal microbial communities among different animals and humans were compared, and the usability of tree shrews as CDI model was further estimated.

## Results

### Clinical features

**Fig. 1:**
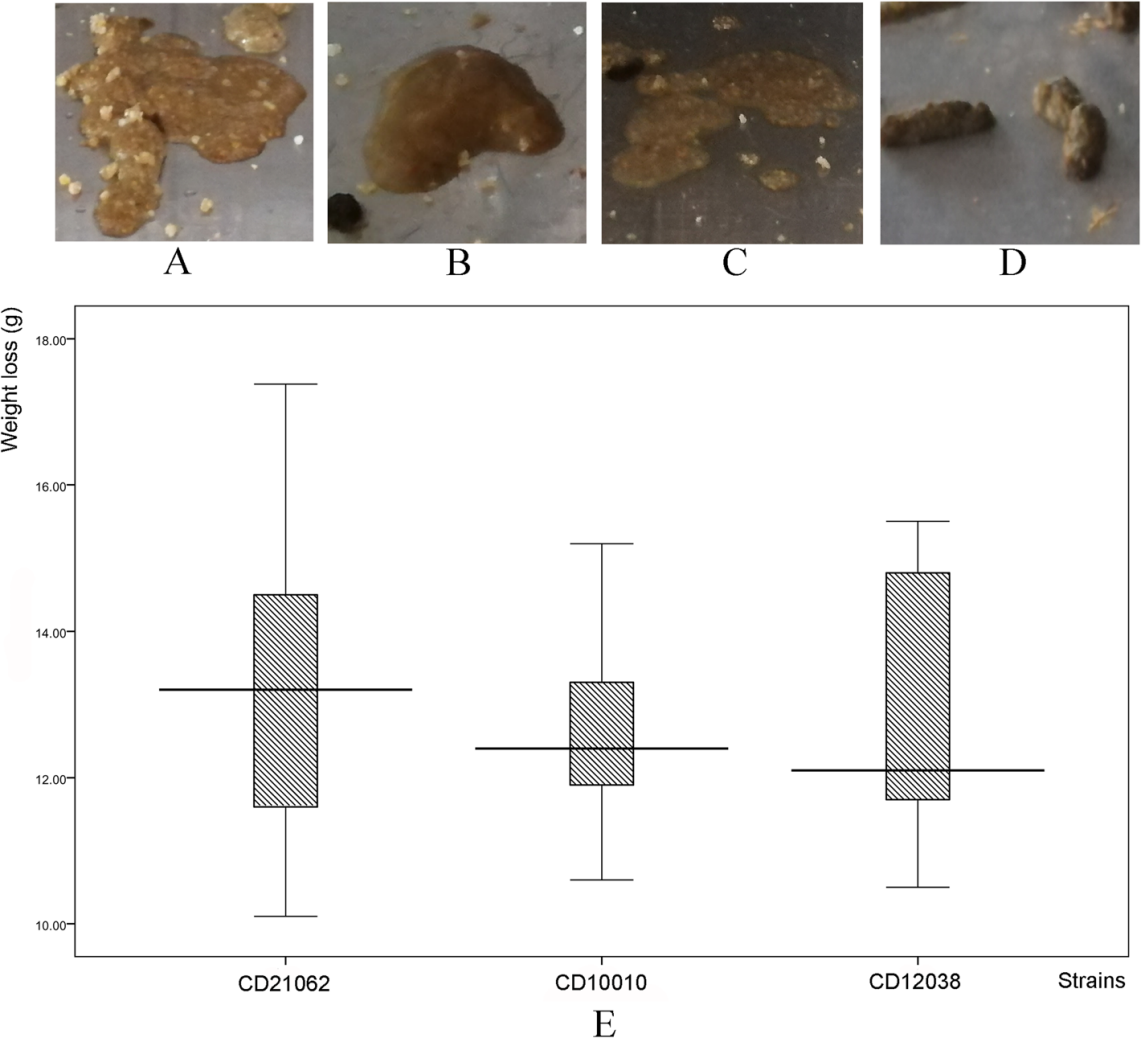
The disease signs of CDI and weight loss of animal in this study. A to C. loose and mucous stools after tree shrew infected with *C. difficile*; D. normal control; E. the weight loss of animals in this study

Nine of 15 infected animals showed clinical signs of CDI, including diarrhea and weight loss (Fig. 1). The remaining animals did not exhibit signs of diseases despite being colonized with *C. difficile*. Four days after infection, the animals began suffering diarrhea (i.e., loose stools containing mucous) (Fig. 1.A to D). There was not a statistically significant difference in weight loss between groups (each strain represented a group) (Fig. 1.E, F = 0.115, *P* = 0.892; average weights 13.35 ± 2.79 g for CD21062, 12.68 ± 1.71 g for CD10010, and 12.92 ± 2.13 g for CD12038). No animals died during the experiment. Fecal samples were all positive for *tcdA*, *tcdB*, *cdtA,* and *cdtB* two days after infection. Fecal samples were toxin negative before infection. The control group for gross anatomy of the abdominal cavity showed normal gut structure of the tree shrew, without any congestion, bleeding or inflammation (Additional file 1.A). However, the infected group of animal indicated the typical inflammation for anatomy of the abdominal cavity, the mesenterium of the tree shrew emerged bled or congestion, and the gut structure disorders could be found (Additional file 1.B).

### Elisa

**Fig. 2:**
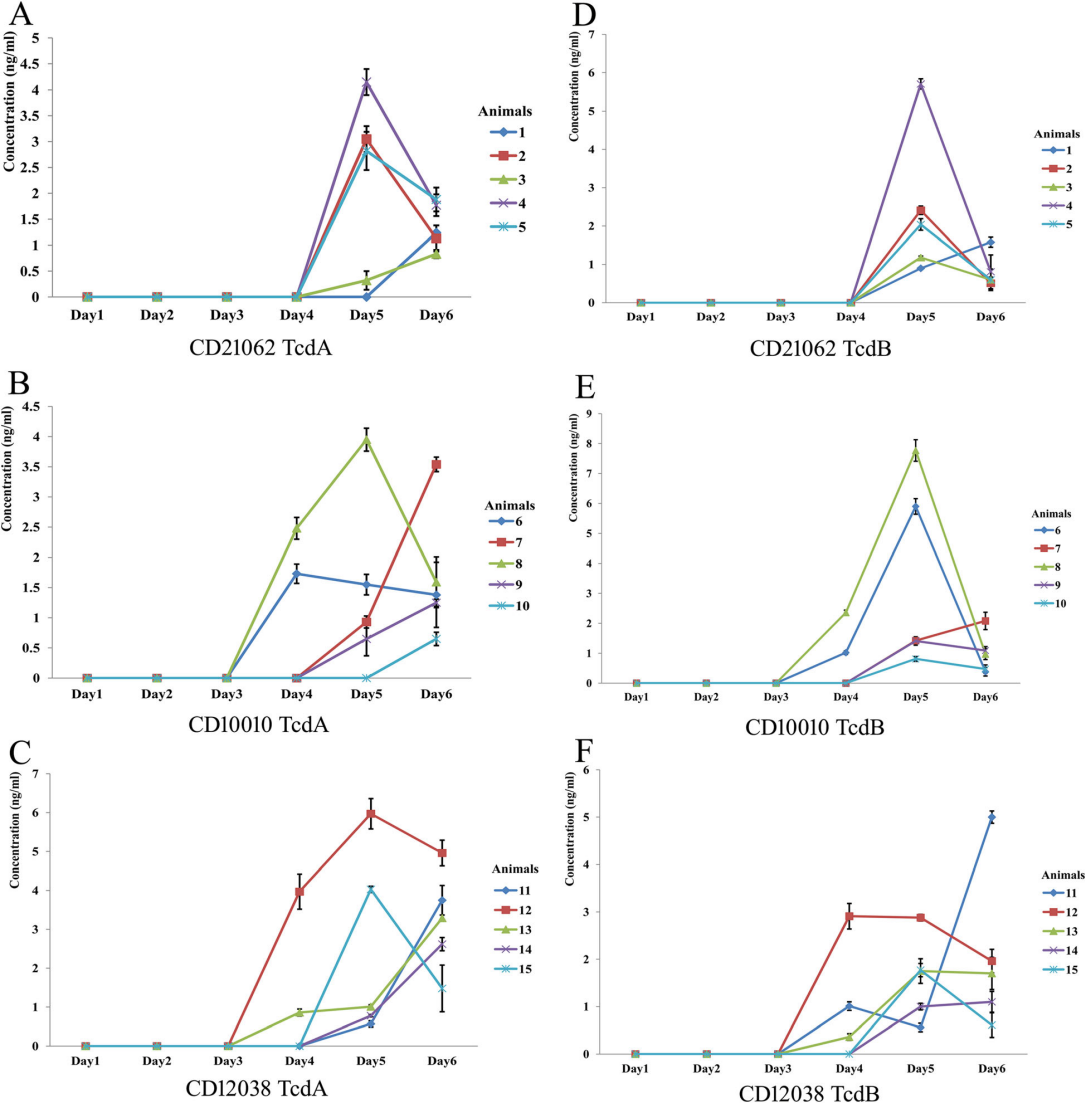
The TcdA and TcdB toxins results of infected tree shrew feces during the experiment. A. TcdA toxin infected by CD21062; B. TcdA toxin infected by CD10010; C. TcdA toxin infected by CD12038; TcdB toxin infected by CD21062; TcdB toxin infected by CD10010; TcdB toxin infected by CD12038

TcdA toxin could be detected at day 4 after oral gavage of the animals, shown as Fig. 2. A-C. CD10010 and CD12038 strain produced TcdA toxin from two tree shrews at day 4, while toxin could be detected at day 5 for CD21062 strain. At experimental day 5, most of the animal feces could be identify the TcdA. Similar results of TcdB toxin were showed from Fig. 2. D-F. At day 5, both TcdA and TcdB reached the higher levels for some of the tree shrews, after that, the concentration of toxins were relatively stable.

### Histopathology

**Fig. 3:**
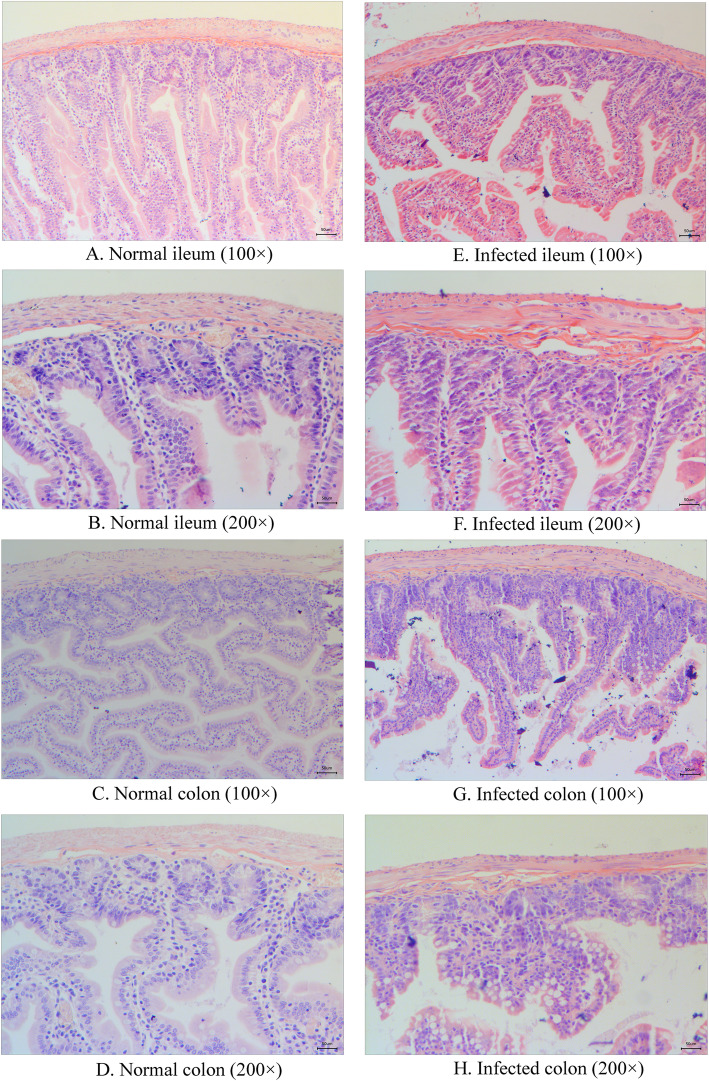
Histopathologic examination of ileum and colon of tree shrew infected with *C. difficile.* A. Normal ileum (100×); B. Normal ileum (200×); C. Normal colon (100×); D. Normal colon (200×); E. infected ileum (100×); F. infected ileum (200×); G. infected colon (100×); H. infected colon (200×)

Histopathology changes were detected in the ileal and colonic tissues of all animals. In healthy ileal and colonic tissues, mucosal cells and structures were arranged neatly and lacked congestion, edema, and inflammatory infiltration (Figs. 3.A to D). Pathology following infection showed evidence of epithelial cell destruction, structural disorders, mucosal ulcers, and necrosis in the mucosa and submucosa of the ileum. Gland and crypt destruction, as well as goblet cell reduction and inflammatory cell infiltration, was observed in mucosal and submucosal layers (Figs. 3.E and F). There was an increase in eosinophil cells, as well as an increase in the infiltration of lymphocyte and plasma cells into colonic tissues in the mucosal and submucosal layers (Figs. 3. G and H).

### Taxonomy of the gut microbiota

3,794,301 reads were obtained from 45 fecal samples. 3,690,628 reads survived merging and quality trimming. The average read length was 423.62 ± 6.93 bp and ranged from 408 to 430 bp. Reads were mapped to 3675 OTUs.

**Fig. 4:**
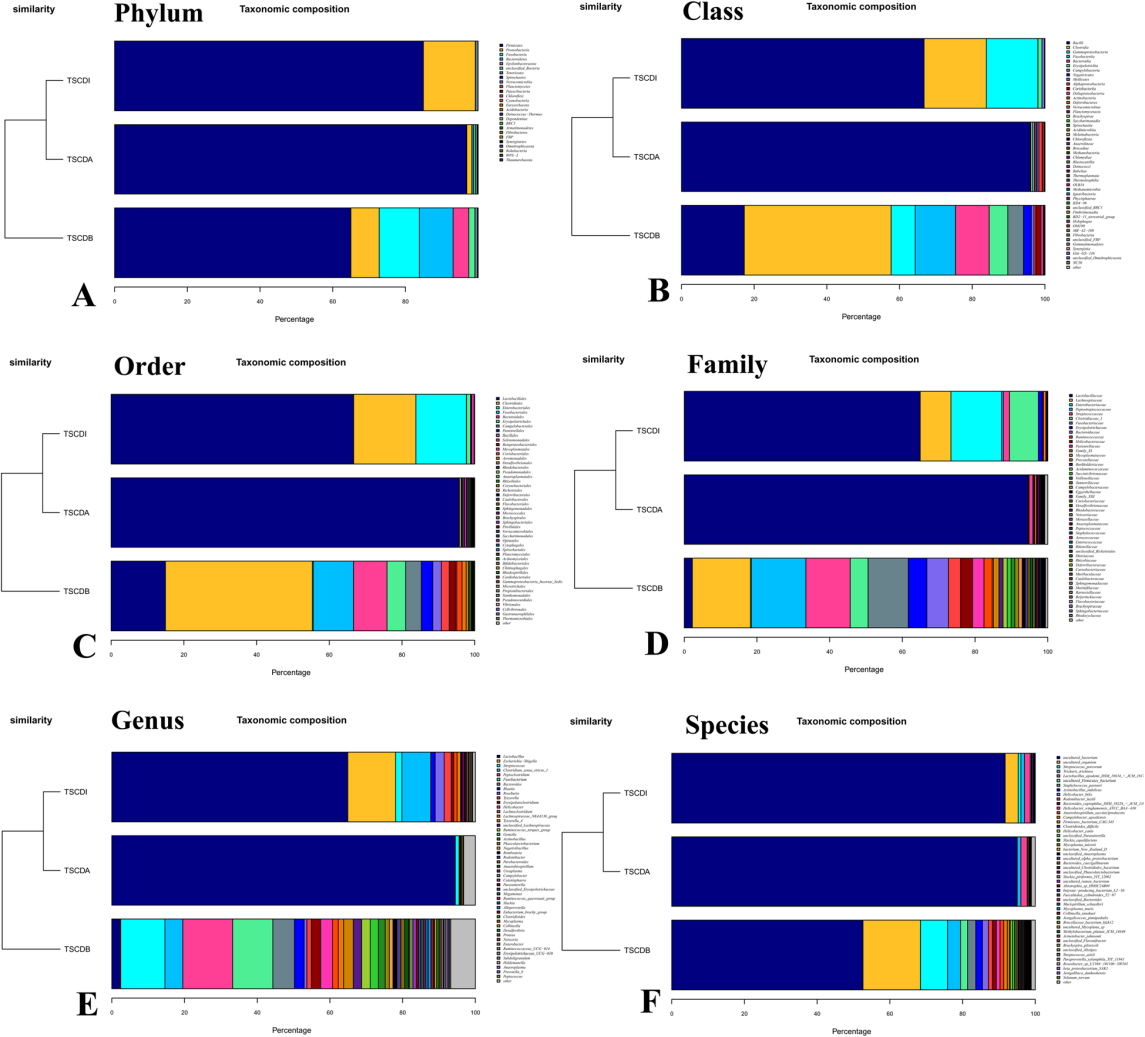
Relative abundance of taxa of tree shrew during the *C. difficile* infection process. A. The relative abundance of taxa at phylum level; B. The relative abundance of taxa at class level; C. The relative abundance of taxa at order level; D. The relative abundance of taxa at family level; E. The relative abundance of taxa at genus level; F. The relative abundance of taxa at species level.

At baseline (TSCDB group), Firmicutes (64.98%), Fusobacteria (11.06%), Bacteroidetes (9.28%), Proteobacteria (7.79%), and Epsilonbacteraeota (4.29%) were the most abundant phyla (Fig. 4.A). *Clostridia* (40.40%), *Bacilli* (17.28%), *Fusobacteriia* (11.06%), *Bacteroidia* (9.28%), and *Gammaproteobacteria* (6.64%) were the most abundant classes (Fig. 4.B); *Clostridiales* (40.40%), *Lactobacillales* (14.93%), *Fusobacteriales* (11.06%), *Bacteroidales* (9.23%), and *Erysipelotrichales* (5.13%) were the most abundant orders (Fig. 4.C). *Lachnospiraceae* (15.99%), *Peptostreptococcaceae* (14.97%), *Streptococcaceae* (12.19%), *Fusobacteriaceae* (11.06%), and *Bacteroidaceae* (5.92%) (Fig. 4.D) were the most abundant families. *Peptoclostridium* (13.73%), *Streptococcus* (12.19%), *Fusobacterium* (11.06%), *Bacteroides* (5.92%), and *Clostridium* (4.89%) were the most abundant genera (Fig. 4.E). *Streptococcus_porcorum* (7.47%), *Trichuris_trichiura* (3.57%), *Staphylococcus_pasteuri* (2.24%), uncultured_Firmicutes_bacterium (1.93%), and *Actinobacillus_indolicus* (1.84%) were the most abundant species (Fig. 4.F).

Antibiotic treatment (TSCDA group) generally decreased the relative abundances of most taxa and caused an outgrowth of *Lactobacillus*. Firmicutes (96.87%), *Bacilli* (96.12%), *Lactobacillales* (95.80%), *Lactobacillaceae* (94.46%), and *Lactobacillus* (94.46%) were the dominant phyla, class, order, family, and genera, respectively (Fig. 4).

The relative abundances of some taxa recovered followed by *C. difficile* infection (TSCDI group), such as Proteobacteria (phylum, 14.28%), *Clostridia* (class, 17.11%), *Gammaproteobacteria* (class, 14.18%); *Clostridiales* (order, 17.11%), *Enterobacteriales* (order, 13.92%); *Enterobacteriaceae* (family, 13.92%), *Lachnospiraceae* (family, 8.45%) and *Clostridiaceae* (family, 7.94%), *Escherichia* (genus, 13.14%), *Clostridium* (genus, 7.94%), *Roseburia* (genus, 2.47%), and *Tyzzerella* (genus, 1.95%) (Fig. 4). The communities during the antibiotic and infection phase were more similar to each other than to the baseline community (Fig. 4).

### Diversity analysis

**Fig. 5:**
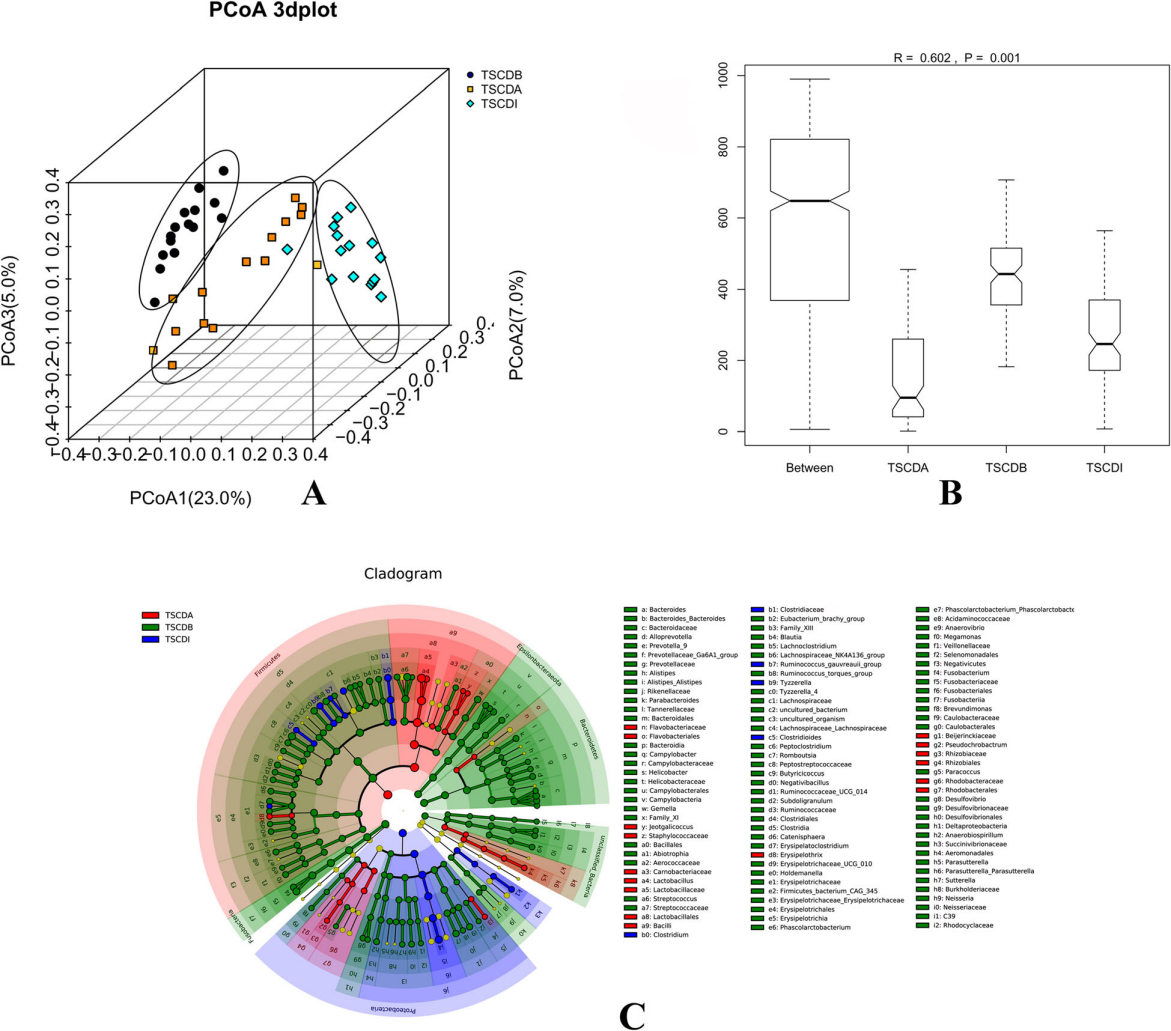
PCoA plot based on Fast UniFrac distance metric and Anosim statistics of three groups. A. PCoA plot based on Fast UniFrac distance; B. Anosim statistics of three groups; C. Cladogram of the phylogenetic distribution of microbial communities associated with the experimental process

There were statistically significant differences in alpha diversity (Richness, Shannon index, ACE index, and Simpson’s index all *P* < 0.05) between TSCDB, TSCDA and TSCDI groups (Table 1). A PCoA plot based on the UniFrac distance matrix revealed clustering by group (Fig. 5.A) and statistically significant differences between groups (Anosim R = 0.602, *P* = 0.001; Fig. 5.B).

**Table 1:** The alpha diversity estimation results in this study

Indexs	Groups	Values (mean ± STD)	min	max	Kruskal Wallis H-test	*P* values
Richness	TSCDB	430.07 ± 62.41	291	535	23.95	0.000
	TSCDA	312.40 ± 200.43	139	847		
	TSCDI	188.40 ± 52.42	111	326		
Shannon entropy	TSCDB	3.24 ± 0.66	2.02	4.15	31.63	0.000
	TSCDA	0.57 ± 0.81	0.04	2.5		
	TSCDI	1.11 ± 0.61	0.17	2.18		
ACE index	TSCDB	851.32 ± 177.61	531.64	1243.92	10.50	0.005
	TSCDA	1130.90 ± 376.96	668.35	1812.25		
	TSCDI	1440.80 ± 699.60	399.31	3065.4		
Chao1 index	TSCDB	657.89 ± 107.66	456.21	824.95	1.768	0.413
	TSCDA	681.25 ± 120.25	444.71	921.43		
	TSCDI	637.96 ± 275.29	229.58	1380		
Simpson’s index	TSCDB	0.11 ± 0.07	0.03	0.22	34.16	0.000
	TSCDA	0.84 ± 0.22	0.3	0.99		
	TSCDI	0.54 ± 0.22	0.22	0.95

A cladogram based on LEfSe analysis showed the phylogenetic distribution of microbial communities in different groups (Fig. 5.C). The dominant taxa at baseline were Firmicutes (*Clostridia*, *Clostridiales* and *Erysipelotrichia*, *Erysipelotrichales*), Bacteroidetes (*Bacteroidia*, *Bacteroidales,* and *Bacteroidaceae*), Fusobacteria (*Fusobacteriia*, *Fusobacteriales,* and *Fusobacteriaceae*) and Epsilonbacteraeota (*Campylobacteria* and *Campylobacterales*). The dominant taxa during antibiotic treatment was *Bacilli* (*Lactobacillales*, *Lactobacillaceae,* and *Lactobacillus)*. After *C. difficile* infection, the relative abundance of *Clostridiales* (*Clostridiaceae* and *Clostridium*), *Ruminococcus,* and *Tyzzerella* increased. The relative abundance of most taxa decreased between antibiotic treatment and infection (Wilcoxon test). In addition to *Clostridium*, the relative abundance of Proteobacteria, *Gammaproteobacteria*, *Enterobacteriales*, *Lachnospiraceae*, *Enterobacteriaceae*, *Escherichia*, *Blautia,* and *Tyzzerella* increased (Table 2). COG and KEGG pathway annotation results based on PICRUSt revealed an increase in the abundance of energy production and conversion; amino acid transport and metabolism; carbohydrate transport and metabolism; transcription; replication, recombination and repair; cell wall/membrane/envelop biogenesis; signal transduction mechanisms; defense mechanisms in COG pathways, and amino acid metabolism; carbohydrate metabolism; cell motility; energy metabolism; infectious diseases; replication and repair; transcription; translation; cellular processes and signaling in KEGG pathways after *C. difficile* infection (TSCDI group) compared to antibiotic treatment (TSCDA group). Functional predictions revealed that bacterial reproduction and metabolism increased after *C. difficile* infection (Additional file 2). However, the abundance of COG and KEGG pathways were highest at baseline in TSCDB group compared with TSCDA and TSCDI group.

**Table 2:** Differential taxa abundance between TSCDA and TSCDI group in this study

	Taxa	Abundance after *C. difficile* infection	P value
Phylum	Proteobacteria	Increase	0.002
	Fusobacteria	Decrease	0.001
	Bacteroidetes	Decrease	0.004
	Epsilonbacteraeota	Decrease	0.002
Class	*Clostridia*	Increase	0.001
	*Gammaproteobacteria*	Increase	0.000
	*Fusobacteriia*	Decrease	0.001
	*Bacteroidia*	Decrease	0.002
	*Campylobacteria*	Decrease	0.002
Order	*Clostridiales*	Increase	0.001
	*Enterobacteriales*	Increase	0.001
	*Fusobacteriales*	Decrease	0.001
	*Bacteroidales*	Decrease	0.003
	*Campylobacterales*	Decrease	0.002
Family	*Lachnospiraceae*	Increase	0.019
	*Enterobacteriaceae*	Increase	0.001
	*Clostridiaceae*	Increase	0.000
	*Fusobacteriaceae*	Decrease	0.001
	*Bacteroidaceae*	Decrease	0.004
	*Helicobacteraceae*	Decrease	0.003
Genus	*Escherichia*	Increase	0.001
	*Clostridium*	Increase	8.77E-05
	*Fusobacterium*	Decrease	0.001
	*Bacteroides*	Increase	0.004
	*Blautia*	Increase	0.028
	*Tyzzerella*	Increase	0.005
	*Helicobacter*	Decrease	0.003

## Discussion

Animal models of human diseases are generally used in the early phases of the understanding of the pathophysiology of a disease, especially when human studies are not feasible for ethical concerns or practical reasons. Although studies on animal models of *C. difficile* infection have been reported, there were still some problems. Thus, the animal model research of *C. difficile* was still necessary. Animal models with high similarities to humans are desirable for studying the pathophysiology and mechanisms of disease [13]. Use of large primates is generally prohibited for ethical reasons. Rodents are commonly used, but these models do not always reflect human pathology [17–19]. Tree shrews are a good model for human biochemical metabolism, physiological function, and genomics [12], and have been widely employed in recent decades. However, this study was the first to investigate *C. difficile* infection in tree shrews. Animals developed diarrhea and weight loss, and histopathology revealed an inflammatory response and structural destruction of the ileum and colon. These results indicated that the tree shrew could be used for CDI studies.

The gut microbiota of mammals is a complex microbial community that plays an important role in maintaining homeostasis [20]. The indigenous gastrointestinal microbiota protects the host from colonization of pathogenic organisms, and many disease processes are linked to the microbiota [21]. Disruption of the gut microbiota is another factor in *C. difficile* pathogenesis, and several CDI risk factors are associated with the microbiota [22]. In this study, we analyzed the intestinal microbial communnity alterations during the *C. difficile* infection in tree shrews, and compared their features. Recent studies have compared the microbiome profiles of human patients with CDI to healthy controls using next generation sequencing, and have shown a decreased diversity and richness in CDI patients [20, 23]. Generally speaking, CDI patients exhibited lower relative abundances of *Lachnospiraceae*, *Bacteroidiaceae,* and *Ruminococcaceae* with corresponding increases in the relative abundances of Proteobacteria, *Lactobacilliaceae*, *Enterococcaceae,* and *Streptococcaceae.* Perez-Cobas et al. [24] showed that *Lactobacillus*, *Enterococcus*, and *Clostridium* were enriched in CDI patients; this was associated with increases in a group of metabolic processes related to amino acids, proteins, and stress response. *Ruminococcaceae*, *Oscillibacteraceae*, *Eubacteriaceae* and *Escherichia* were identified as taxa exhibiting potential for *C. difficile* colonization resistance. We found that the response of tree shrew to CDI was similar to that of humans. The relative abundance of many taxa significantly decreased after antibiotic administration and *C. difficile* infection, including Fusobacteria, Bacteroidetes, and Epsilonbacteraeota. The relative abundance of *Fusobacteriaceae*, *Bacteroidaceae,* and *Helicobacteraceae* were lower during infection than during antibiotic treatment; meanwhile the relative abundance of *Lachnospiraceae*, *Enterobacteriaceae*, *Escherichia, Bacteroides* and *Clostridium* were higher. *Ruminococcaceae* and *Eubacteriaceae* showed similar tendencies but were not statistically significant. In addition, the relative abundance of *Blautia* and *Tyzzerella* increased after CDI. Consequently, we considered that *Lachnospiraceae*, *Enterobacteriaceae*, *Bacteroides*, *Escherichia*, *Blautia,* and *Tyzzerella* could be used as microbial marker for *C. difficile* infection in tree shrew model. Some of the taxa, such as *Enterobacteriaceae* and *Escherichia* were commonly increased both in human and animals after *C. difficile* infection, so these taxa exhibited resistance for *C. difficile* colonization generally. Besides, the metabolic pathways during the infection process of tree shrew also indicated that diversity of the gut microbiota was important for *C. difficile* infection. Before antibiotic treatment and spores gavage, large diversity was found in gastrointestinal tract of tree shrew, and the functional prediction revealed the highest abundance in TSCDB group. After antibiotics usage, the diversity was decreased in TSCDA group, and the abundance of all the COG and KEGG pathways were lowest at this stage, such as energy metabolism, amino acid and carbohydrate metabolism, replication, transcription and defense mechanisms. Thus, the host perhaps was susceptible to pathogen. After *C. difficile* infection, some of the abundance of metabolic pathways increased, and part of the microbial marker could be identified as mentioned before, and these taxa probably were resistant for *C. difficile* infection.

The increase in relative abundance of *Lactobacillus* was also interesting. *Lactobacillaceae* was dominant in CDI samples from both humans and mice [25, 26]. In humans, the presence of *Lactobacillus* in the intestinal microbiota was generally considered an index of good health and not a trigger for *C. difficile* infection. However, *Lactobacillus* appeared to be resistant to the antibiotics used in this study. Maybe *C. difficile* infection was favored by a global decrease of alpha diversity, and the overgrowth of *Lactobacillus* might represent an epiphenomenon due to reduced competition for ecological niche. *Lactobacillus* remained the dominant taxa after CDI; however, some taxa, such as *Escherichia*, *Streptococcus*, *Clostridium*, *Roseburia,* and *Tyzzerella,* did recover*.*

Reeves et al. [27] used murine model of CDI to demonstrate the changes in endogenous microbial communities against CDI and found a significant change in the structure of the gut microbial community. Antibiotic administration resulted in an outgrowth of *Lactobacillaceae*. Animals developing clinical symptoms had gut microbiota that were dominated by Proteobacteria. Our results showed similar trends, that was the similar gut microbiota changes during the CDI between murine and tree shrew model. We further summarized the characteristics of *C. difficile* infections among tree shrew, rodent animals and human patients (Table 3). In this study, the infection method and antibiotics administration were based on previous study, which used the rodents as the animal model. The typical symptoms or signs of diseases were similar between tree shrew and rodents, such as diarrhea or weight loss; however, there was no death of tree shrew. For lesion site of infection, both tree shrew and rodents showed some differences with human patients, as Table 3 shown. In addition, the gut microbiota changes during the infection process were similar among them; the microbial communities of tree shrew, rodents and human were similar before infection, during the antibiotics treatments and even after the infection. Some microbial taxa could be used as commonly *C. difficile* colonization biomarkers, such as Proteobacteria, *Enterobacteriaceae* and *Escherichia.* Therefore, the tree shrew appeared to be a useful model of for CDI, and further studies were warranted.

**Table 3:** Comparison of the features for *C. difficile* infection among tree shrew, rodents and human

		Tree shrew	Rodents (hamster or mouse)	Human	References
Infective dose		10^5^CFU spores	10^5−^ 10^9^ CFU spores or vegetative	–	9, 19, 27
Routes of infection		Oral gavage	Oral gavage	Fecal-oral transmission	9, 19, 27
Antibiotics administration		Antibiotic cocktails	Clindamycin treated; Antibiotic cocktails; Cefoperazone treated	Antibiotics exposure	9, 17, 18, 19, 27
Disease features	Numbers of infection	60% (9/15) exhibited signs of disease at 4 days	All hamsters developed diseases; parts of the mouse (58%) showed the clinical signs of CDI at 2 to 4 days	Usually happened in elderly patients or antibiotics usage	9, 27
	Symptoms or signs	Diarrhea; weight loss	Wet tail; diarrhea; weight loss	From asymptomatic to severe colitis	9, 18, 19, 27
	Deaths	None	Most died within 48 h for hamster; few death occurred for mouse	Some cases developed to death	9, 17, 18
Lesion sites		Ileum and colon	Colon and cecum	Colon	9, 17, 18
Gut microbiota changes	Normal or before infection	Dominated with Firmicutes, Fusobacteria, Bacteroidetes and Proteobacteria phylum	Dominated with Firmicutes and Bacteroidetes phylum	Dominated with Firmicutes, Proteobacteria and Bacteroidetes phylum	18, 19, 20, 23, 24, 27
	Antibiotics treatments	Lower microbial diversity and richness; dominated with *Lactobacillus*	Lower microbial diversity and richness; dominated with *Lactobacillus*	Lower microbial diversity and richness; dominated with *Lactobacillus*	18, 19, 20, 23, 24, 27
	After *C. difficile* infection	Proteobacteria, *Clostridiales*, *Enterobacteriaceae*, *Lachnospiraceae* and *Escherichia* increased	Proteobacteria, *Enterobacteriaceae*, *Verrucomicrobiaceae* and *Lachnospiraceae* increased	Bacteroidetes, *Lachnospiraceae*, *Enterobacteriaceae*, and *Escherichia* increased	18, 19, 20, 23, 24, 27

## Conclusions

The tree shrew is a novel used laboratory animal. Use of the tree shrew as a laboratory animal has increased in recent years due to its low cost of maintenance, life span, small body size, short reproductive cycle, and close relationship to primates. We infected tree shrew with pathogenic *C. difficile* strains. The disease manifestations, histopathology of infections were similar with human and other animal models. The gut microbiota changes during the infection process reflected the important role of gut microbial community in this animal, and mimicked those in humans. All these pieces of evidences indicated tree shrew can be used as a novel animal model for *C. difficile* infection study.

## Methods

### Animal sources

Twenty tree shrews were used for this study, which was conducted at the Center of Tree Shrew Germplasm Resources, Institute of Medical Biology, Chinese Academy of Medical Science and Peking Union Medical College in Kunming, China. The tree shrews were from a closed population, and were healthy animals without visible signs of disease or tumors. Ten of the animals were male and 10 were female. The average age was 13.0 ± 1.5 months, and ranged from 11 to 15 months. We divided these animals into four groups that were infected with the CD21062, CD10010, or CD12038 strain of *C. difficile* and one control group without any administration. Each group contained five animals. All of the tree shrews used in this study were the first filial generation and weighed 146.49 ± 14.60 g. Fecal samples were collected at baseline, during antibiotic treatment, and after *C. difficile* infection. Briefly, fecal samples were collected before antibiotics usage, defined as TSCDB group; feces were collected after antibiotics feeding at day 6, defined as TSCDA group; day 5 of *C. difficile* infection in tree shrew was define as TSCDI group. Total genomic DNA was extracted using a fecal DNA extraction kit (Tiangen, Beijing) according to the manufacturer’s instructions [28]. DNA samples were stored at -20 °C until they were used for 16S rRNA gene sequencing and virulent gene (*tcdA*, *tcdB*, *cdtA,* and *cdtB*) detection [2].

### Bacterial source and spore preparation

The CD21062, CD10010, and CD12038 strains were all isolated from elderly patients in a tertiary hospital in Beijing, China, and have been previously described [8]. All these strains belonged to ST11, CD21062 was RT078, and others were new ribotypes. All three strains tested positive for the toxin genes *tcdA*, *tcdB*, *cdtA*, and *cdtB*, and the genomes of the strains were deposited in the NCBI database (accession PRJNA497978). The strains were cultured on brain heart infusion (BHI) agar containing 5% cysteine. An anaerobic environment was maintained at all times using an anaerobic chamber (MITSUBISHI, Japan). An incubation temperature of 37 °C was used for *C. difficile* growth.

To prepare spores, strains were incubated under anaerobic conditions for seven days. All surface growth (containing vegetative cells, debris and spores) was extracted and transferred to microcentrifuge tubes containing 1 mL of sterile ice-cold water. The mixtures were centrifuged five times for 1 min at 13,000×g. The washed pellets were suspended in 833 μL of 20% HistoDenz (Sigma-Aldrich) and combined into a 5 mL mixture. The suspension was gently layered onto 10 mL of 50% HistoDenz in a 15 mL centrifuge tube and centrifuged for 15 min at 15,000×g at 4 °C. The spores were resuspended in 1 mL of sterile ice-cold water, and centrifuged for 1 min at 13,000×g. The procedure was repeated five times to remove HistoDenz, and final pellet was resuspended in 200 μL of water [29].

### Antibiotic treatment and infection with *C. difficile*

Tree shrews were housed in 20 sterile cages containing irradiated food and autoclaved water. The experimental animals were received an antibiotic pretreatment, which has been previously described [30]. In general, tree shrews received the antibiotic cocktail for 7 days in the drinking water [metronidazole (0.215 mg/mL), vancomycin (0.045 mg/mL), kanamycin (0.4 mg/mL), gentamicin (0.035 mg/mL) and colistin (850 U/mL)]. A single intraperitoneal injection of clindamycin (10 mg/kg) was administered one day prior to infection. Tree shrews were then infected by oral gavage of 10^5^ spores of *C. difficile*. The animals were observed daily for signs of disease (i.e., diarrhea or ruffled fur). The tree shrews were euthanized seven days after gavage and the gross anatomy of the abdominal cavities were compared between infected and control ones. The animal intestinal tissues were collected following anesthesia and euthanasia method: tree shrews were cervical dislocated 5 min following an intraperitoneal injection of 2% pentobarbital sodium (0.2 ml/100 g, Sigma-Aldrich, USA). Ileal and colonic tissues were collected, washed with PBS, and cut in five pieces for further analyses.

### Elisa

TcdA and TcdB toxins of the infected tree shrew stools were detected by ELISA method. The fresh fecal samples were collected after *C. difficile* infection for one week of all animals. Feces were suspended to 0.5 g/ml concentration by using PBS buffer (pH 7.4). The fecal suspensions were coated in 96-well plate at 4 °C overnight, and then washed with PBST (PBS pH 7.4 + 0.05% tween-20). The plate was blocked with 5% skim milk at 37 °C for 2 h. The polyclonal antibodies of TcdA and TcdB (List Biological Laboratories, Chicken IgY, 1: 1000 dilution) were used for first hybridization; then Goat anti-Chicken IgY Secondary Antibody, HRP (Invitrogen, 1: 5000 dilution) was used. ELISA-TMB Chromogenic Reagent kit (Sangon, China) was used to determine the absorbance at 450 nm. Pure toxin A and toxin B (List Biological Laboratories) were used as positive controls, and the standard curves were determined at 20 ng/ml, 2 ng/ml, 0.2 ng/ml, 0.02 ng/ml and 0.002 ng/ml both for TcdA and TcdB.

### Histopathology

Histopathology was performed to evaluate inflammation and mucosal damage. Resected ileal and colonic tissues were fixed in 4% paraformaldehyde and stained with hematoxylin-eosin staining (HE) for histological analysis [23].

### PCR amplification, library construction, and sequencing

The V3 to V4 variable region of the 16S rRNA gene was amplified using previously described primers [31]. Library preparation followed guidelines from Illumina. PCR was performed using the KAPA HiFi HotStart ReadyMix kit (Kapa, Biosystems). The amplification procedure has been previously described. PCR products were purified with AMPure XP magnetic beads (Beckman, Coulter) and quantified using a Qubit fluorometer (Invitrogen, Life Technologies). Secondary PCR amplification was performed to add Illumina Nextera barcodes and the products we purified to remove nontarget fragments. Amplicons were normalized, pooled, and sequencing using an Illumina Miseq system (Illumina, San Diego, USA) [28].

### Bioinformatics and statistical analysis

The raw data were trimmed and low quality (<Q25) reads were removed. The paired end reads were merged and barcodes were removed using PEAR 0.9.6, cutadapt 1.2.1, and Prinseq 0.20.4 [32, 33]. QIIME 2.0, USEARCH 11.0, and R 3.2 were used for bioinformatics analysis [34–36]. Sequences were clustered into operational taxonomic units (OTUs) according to 97% sequence similarity against the Silva 132 database using the UPARSE pipeline. OTUs were named using SILVA taxonomic nomenclature [37]. Principal coordinate analysis (PCoA) was used to visualize the similarities between the three groups. Analysis of similarities (Anosim) was used to compare the differences in microbial communities between groups. Linear discriminant analysis effect size (LEfSe) analysis was used to identify bacteria with statistically significant (*P* < 0.05) differences in abundance between groups. Phylogenetic investigation of communities by reconstruction of unobserved states (PICRUSt) was used to predict the functional potential of the communities. Statistical analyses were performed using SPSS (version 16.0, IBM, USA). The Kolmogorov-Smirnov test, T-test, or Kruskal-Wallis H test were used as appropriate. A *P*-value < 0.05 was considered statistical significance. Sequence data were deposited to the NCBI database (SRA accession: PRJNA541587).

## Supplementary information

**Additional file 1.** The gross anatomy of the abdominal cavities between infected and control tree shrew. A. Normal control; B. infected animal.

**Additional file 2.** COG and KEGG pathway annotation results based on PICRUSt.

## Data Availability

The datasets generated and/or analysed during the current study are available in the NCBI database repository by the SRA accession: PRJNA541587, [https://www.ncbi.nlm.nih.gov/bioproject/PRJNA541587].

## Abbreviations

CDI: *Clostridioides difficile* infection
BHI: brain heart infusion
OTUs: operational taxonomic units.
PCoA: Principal co-ordinates analysis
LEfSe: Linear discriminant analysis Effect Size
PICRUSt: Phylogenetic investigation of communities by reconstruction of unobserved states
