# Supplementary figures and images for: Response of the gut microbiota during the Clostridioides difficile infection in tree shrews mimics those in humans
Source: BMC Microbiol. 2020 Aug 20;20:260. doi: 10.1186/s12866-020-01943-z (PMC7441558; doi:10.1186/s12866-020-01943-z)

A

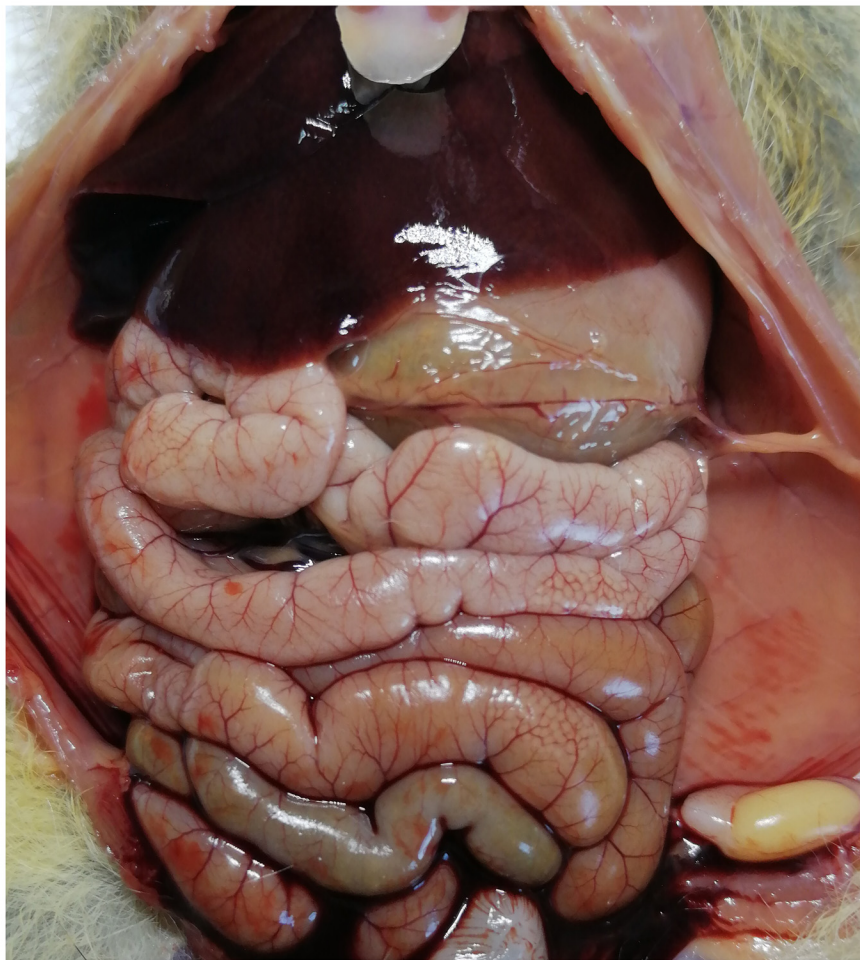

Normal control

B

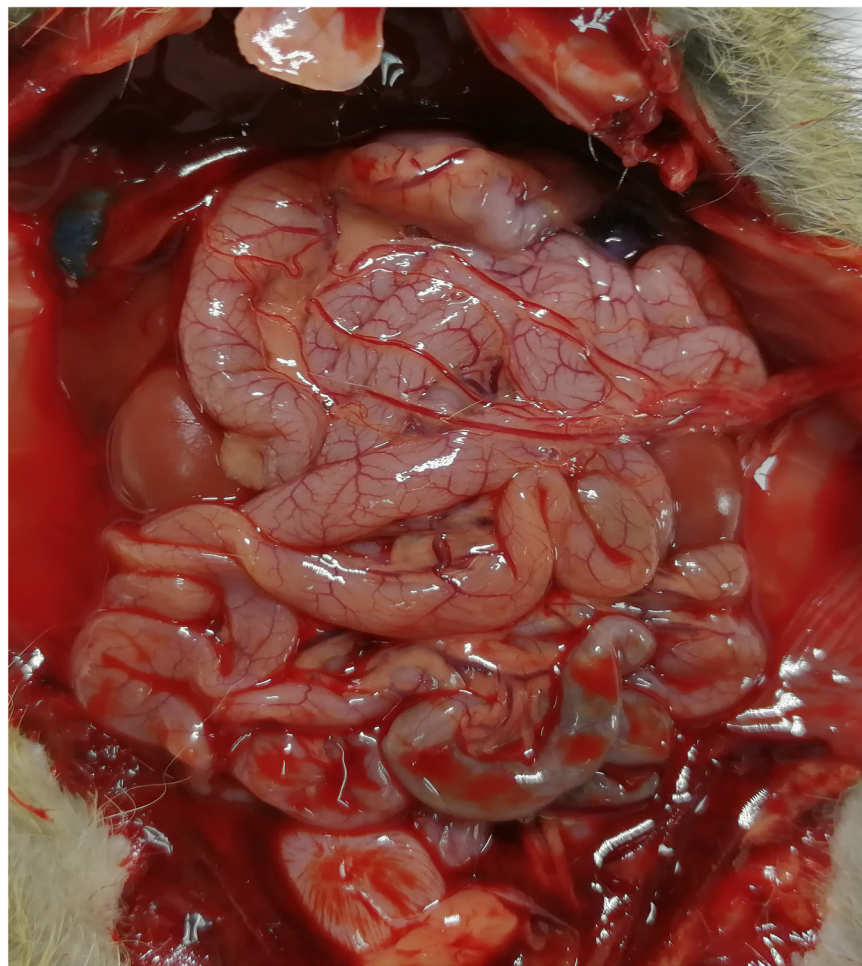

Infected tree shrew

Supplement: Supplementary file 1 — Additional file 1. The gross anatomy of the abdominal cavities between infected and control tree shrew. A. Normal control; B. infected animal. [file 12866_2020_1943_MOESM1_ESM.pdf]

# heatmap of COG

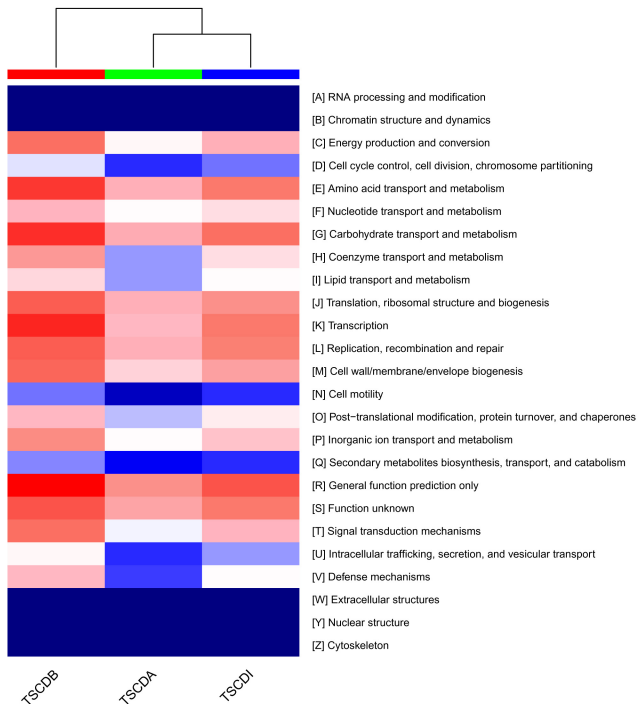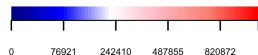

# heatmap of KEGG

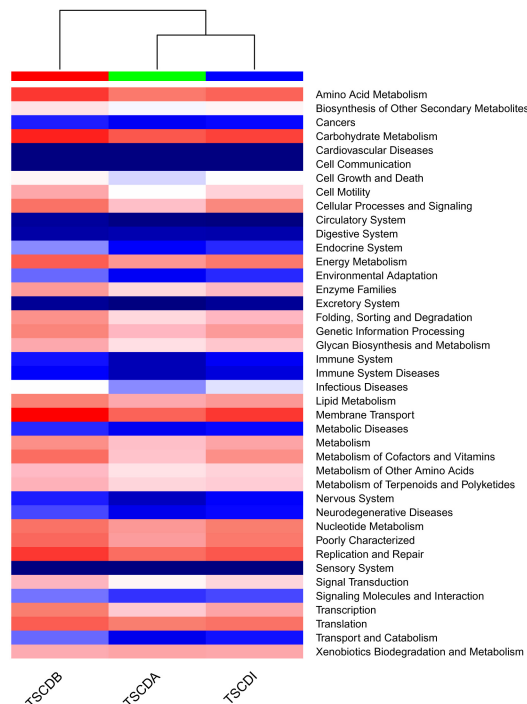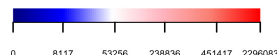

Supplement: Supplementary file 2 — Additional file 2. COG and KEGG pathway annotation results based on PICRUSt. [file 12866_2020_1943_MOESM2_ESM.pdf]
